# Sex differences in hepatitis A incidence rates–a multi-year pooled-analysis based on national data from nine high-income countries

**DOI:** 10.1371/journal.pone.0287008

**Published:** 2023-06-13

**Authors:** Manfred S. Green, Naama Schwartz, Victoria Peer

**Affiliations:** School of Public Health, University of Haifa, Haifa, Israel; Universitair Kinderziekenhuis Koningin Fabiola: Hopital Universitaire des Enfants Reine Fabiola, BELGIUM

## Abstract

**Background:**

Possible sex differences in hepatitis A virus (HAV) incidence rates in different age groups are not well documented. We aimed to obtain stable pooled estimates of such differences based on data from a number of high-income countries.

**Methods:**

We obtained data on incident cases of HAV by sex and age group over a period of 6–25 years from nine countries: Australia, Canada, Czech Republic, Finland, Germany, Israel, Netherland, New Zealand and Spain. Male to female incidence rate ratios (IRR) were computed for each year, by country and age group. For each age group, we used meta-analytic methods to combine the IRRs. Meta-regression was conducted to estimate the effects of age, country, and time period on the IRR.

**Results:**

A male excess in incidence rates was consistently observed in all age groups, although in the youngest and oldest age groups, where the numbers tended to be lower, the lower bounds of the 95% confidence intervals for the IRRs were less than one. In the age groups <1, 1–4, 5–9, 10–14, 15–44, 45–64 and 65+, the pooled IRRs (with 95% CI) over countries and time periods were 1.18 (0.94,1.48), 1.22 (1.16,1.29), 1.07 (1.03,1.11), 1.09 (1.04,1.14), 1.46 (1.30,1.64), 1.32 (1.15,1.51) and 1.10 (0.99,1.23) respectively.

**Conclusions:**

The excess HAV incidence rates in young males, pooled over a number of countries, suggest that the sex differences are likely to be due at least in part to physiological and biological differences and not just behavioral factors. At older ages, differential exposure plays an important role. These findings, seen in the context of the excess incidence rates in young males for many other infectious diseases, can provide further keys to the mechanisms of the infection.

## Introduction

There is an expanding literature on sex differences in the incidence rates of various infectious diseases [1–3]. The type and extent of the differences frequently vary by disease and age group. The mechanisms underlying these differences have not been fully elucidated and cannot be explained entirely by differences in exposure. The pattern of male to female ratios in the incidence rates of different infectious diseases can make an important contribution to understanding the underlying mechanisms of the diseases.

Despite the availability of an effective vaccine, hepatitis A virus (HAV) infection remains a common disease, particularly in low-income countries with overcrowding and poor sanitation, where the incidence rates of the disease are particularly high in infancy and childhood [4, 5]. In countries with high hepatitis A vaccine coverage, the incidence of cases and outbreaks have decreased in children and the infection has shifted significantly to other risk groups, such as men who have sex with men (MSM) [6–9].

There are reports in the literature on sex differences in the incidence rates of hepatitis A, but they are inconsistent and poorly documented by age group [10–13]. While some report higher incidence rates of viral hepatitis A in males [6, 10], there are inconsistencies. For example, in a report from Germany, during 2018–2020, no sex differences were observed in the incidence of the disease [11]. One report from South Korea found a change in the sex differences, possibly due to increased immunization in the military [13].

In this study, we aimed to obtain pooled estimates of the age-specific male to female ratios in the incidence rates of HAV infection based on data from a number of developed countries over extended time periods.

## Methods

### Source of data

National surveillance data on reported cases of HAV infection, by age, sex and year, were obtained from relevant government institutions for nine countries from Czech Republic, Finland, Germany, Netherland, Spain, Australia, New Zealand, Canada and Israel. The data for Australia, for years 2001–2016, was extracted from the National Notifiable Diseases Surveillance System (NNDSS), [14] for Canada for the years 1991–2015, from the Public Health Agency of Canada (PHAC) [15], for the Czech Republic, for 2008–2013, from the Institute of Health Information and Statistics [16], for Finland, for years1995-2016 from the National Institute for Health and Welfare (THL) [17], for Germany for the years 2001–2016, from the German Federal Health Monitoring System [18], for Israel from the Department of Epidemiology in the Ministry of Health for years 1998–2016, for the Netherland (2003–2017), directly from the official representative of RIVM, for New Zealand for years 1997–2015 from the Institute of Environmental Science and Research (ESR) [19] and for Spain from the Spanish Epidemiological Surveillance for years 2005–2015 [20].

Information about the population size by age, sex and year was obtained for Australia from ABS.Stat [21] (Australia’s Bureau of statistics), for Canada from Statistics Canada CANSIM database [22], for the Czech Republic from the Czech Statistical Office [23], for Finland from Statistics Finland’s PX-Web databases [24], for Germany from the German Federal Health Monitoring System [25], for Israel from the Central Bureau of Statistics [26], for Netherland from Netherlands’ database (StatLine) [27], for New Zealand from Statistics New Zealand [28] and for the Spain from the Department of Economic and Social Affairs, Population Division [29].

### Ethics and informed consent

National, open access, sex-and-age disaggregated, anonymous data were used and there was no need for ethics committee approval.

### Statistical analyses

#### Data analysis

HAV incidence rates (IR) per 100,000 were calculated by age group and sex, for each country and calendar year using the number of reported cases divided by the respective population size and multiplied by 100,000. The age groups considered were <1 years (infants), 1–4 (early childhood), 5–9 (late childhood), 10–14 (puberty), 15–44 (young adulthood), 45–64 (middle adulthood) and 65+ (senior adulthood). Surveillance systems in Canada and New Zealand used similar age-groups except for 15–39, 40–59 and 60+. For Australia, data for infants and age 1–4, disaggregated by sex and age, are missing. The male to female incidence rate ratio (IRR) was calculated by dividing the incidence rate in males by that of females, by age group, country and time period.

#### Pooled analysis

As in previous studies of sex differences in infectious diseases [1–3], we used meta-analytic methods to establish the magnitude of the pooled sex differences in the incidence of HAV infection, by age group, across different countries and over a number of years. The outcome variable was the male to female IRR. For each age group, the IRRs for each country were pooled over time periods and then the pooled IRRs for each country were combined. Forest plots with the pooled IRRs, over countries and years of reporting, were prepared separately for the seven age groups. Heterogeneity was evaluated using the Q statistic and I^2^ was calculated as an estimate of the percentage of between-study variance. If the p-value for the Q statistic was less than 0.05, or I^2^ exceeded 50%, the random effects models was used to estimate pooled IRRs and 95% confidence intervals (CI). Otherwise, the fixed effects model was considered, although due to the low power of the Q statistic, the more conservative random effects model was preferred. In order to explore the contribution of countries and the reported years to the variability in the IRRs, meta-regression analyses were performed. To evaluate the effect of individual countries and years on the male to female incidence risk ratio, we performed leave-one-out sensitivity analysis and recomputed the pooled IRRs. The meta-analytic methods and meta-regressions were carried out using STATA software version 12.1 (Stata Corp., College Station, TX).

## Results

### Descriptive statistics

The summary of the male to female IRRs per 100,000 populations in different countries for each age group is presented in Table 1.

**Table 1:** Details of the countries included in the study, by sex and age group—descriptive data.

			Males		Females		
Age	Country	Years	n/N	IR	n/N	IR	RR
**<1**	Canada	1991–2015	29/4682619	0.62	32/4446799	0.72	0.86
	Czech Republic	2008–2013	25/349195	7.16	23/332712	6.91	1.04
	Germany	2001–2016	27/5740478	0.47	23/5448550	0.42	1.11
	Israel	1998–2016	38/1486100	2.56	34/1410400	2.41	1.06
	Netherland	2003–2017	3/1616870	0.19	3/1540059	0.19	0.95
	New Zealand	1997–2015	3/576900	0.52	1/548520	0.18	2.85
	Spain	2005–2015	41/2679186	1.53	17/2514548	0.68	2.26
**1–4**	Canada	1991–2015	679/19156418	3.54	560/18225737	3.07	1.15
	Czech Republic	2008–2013	267/1410748	18.93	222/1343670	16.52	1.15
	Germany	2001–2016	615/23509315	2.62	511/22311030	2.92	1.14
	Israel	1998–2016	778/5731500	13.57	550/5443300	10.10	1.34
	Netherland	2003–2017	94/5811264	1.62	88/5543773	1.59	1.02
	New Zealand	1997–2015	80/2308880	3.46	55/2191980	2.51	1.38
	Spain	2005–2015	587/10880587	5.39	419/10233932	4.09	1.32
**5–9**	Australia	2001–2016	258/11398585	2.26	212/10814642	1.96	1.15
	Canada	1991–2015	1556/24668602	6.31	1506/23469919	6.42	0.98
	Czech Republic	2008–2013	256/1532669	16.70	220/1450621	15.17	1.10
	Finland	1995–2016	42/3440956	1.22	42/3297629	1.27	0.96
	Germany	2001–2016	1361/30760941	4.42	1196/29187252	4.10	1.08
	Israel	1998–2016	1263/6616300	19.09	1012/6287700	16.09	1.19
	Netherlands	2003–2017	208/7478265	2.78	243/7139402	3.40	0.82
	New Zealand	1997–2015	126/2899540	4.35	139/2752910	5.05	0.86
	Spain	2005–2005	983/13017097	7.55	821/12287011	6.68	1.13
**10–14**	Australia	2001–2016	190/11377822	1.67	144/10797396	1.33	1.25
	Canada	1991–2015	954/25685783	3.71	854/24391864	3.50	1.06
	Czech Republic	2008–2013	157/1416001	11.09	148/1339518	11.05	1.00
	Finland	1995–2016	45/3522497	1.28	42/3375446	1.24	1.03
	Germany	2001–2016	908/33455166	2.71	846/31724889	2.67	1.02
	Israel	1998–2016	522/6106400	8.55	430/5807300	7.40	1.15
	Netherlands	2003–2017	182/7658243	2.38	151/7312854	2.06	1.15
	New Zealand	1997–2015	72/2919850	2.47	80/2776650	2.88	0.86
	Spain	2005–2005	535/12301238	4.35	409/11627137	3.52	1.24
**15–44**	Australia	2001–2016	1462/73591102	1.99	990/72741755	1.36	1.46
	Canada	1991–2015	7148/143987472	4.96	3620/140453550	2.58	1.93
	Czech Republic	2008–2013	1428/13725818	10.40	918/12978912	7.07	1.47
	Finland	1995–2016	562/18898064	2.97	332/18050351	1.84	1.62
	Germany	2001–2016	3840/257895408	1.49	2753/247590330	1.11	1.34
	Israel	1998–2016	1103/29586200	3.73	878/29264100	3.00	1.24
	Netherlands	2003–2017	774/39930903	1.94	414/39138712	1.06	1.83
	New Zealand	1997–2015	401/13546700	2.96	295/13976900	2.11	1.40
	Spain	2005–2005	4962/110542308	4.49	2425/105413400	2.30	1.95
**45–64**	Australia	2001–2016	420/41988401	1.00	330/42573071	0.78	1.29
	Canada	1991–2015	2419/110461323	2.19	1390/109655649	1.27	1.73
	Czech Republic	2008–2013	356/8403729	4.24	347/8624880	4.02	1.05
	Finland	1995–2016	220/16513241	1.33	177/16307550	1.09	1.23
	Germany	2001–2016	1742/181698132	15.55	1694/181849520	15.08	1.03
	Israel	1998–2016	140/12368500	1.13	129/13327000	0.97	1.17
	Netherlands	2003–2017	400/36361477	1.10	211/35859166	0.59	1.87
	New Zealand	1997–2015	186/10201030	1.82	126/10685350	1.18	1.55
	Spain	2005–2005	601/63103755	0.95	432/64340310	0.67	1.42
**65+**	Australia	2001–2016	120/21417772	0.56	151/25538457	0.59	0.95
	Canada	1991–2015	693/64590224	1.07	816/78346403	1.04	1.03
	Czech Republic	2008–2013	50/4087584	1.22	93/5999018	1.55	0.79
	Finland	1995–2016	75/11159619	0.67	74/15066114	0.49	1.37
	Germany	2001–2016	948/108019284	0.88	1435/149862231	0.96	0.92
	Israel	1998–2016	65/6010700	1.08	54/7903600	0.68	1.58
	Netherlands	2003–2017	95/24551738	0.39	89/29339281	0.30	1.28
	New Zealand	1997–2015	75/6302700	1.19	70/7386000	0.95	1.26
	Spain	2005–2005	131/37127234	0.35	133/49879431	0.27	1.32

IR = incidence rate, IR per 100 000 Male or Female population, incidence RR = female: male incidence Rate Ratio

n- Cumulative total of cases for given years.

N- Cumulative total of the population for given years.

Infants = age<1 year; early childhood = 1–4 years; late childhood = 5–9 years; puberty = 10–14 years; young adulthood = 15–44 or 15–39 years; middle adulthood = 40–59 or 45–64 years; senior adulthood = 60+ or 65+ years.

Significant differences in incidence rates were observed between the countries, with the highest incidence rates in all ages and both sexes in Czech Republic. Higher incidence rates were observed in Israel and Spain up to age 44 and in Germany in the group of adults (age 45–64).

#### Forest plots

**Fig 1:**
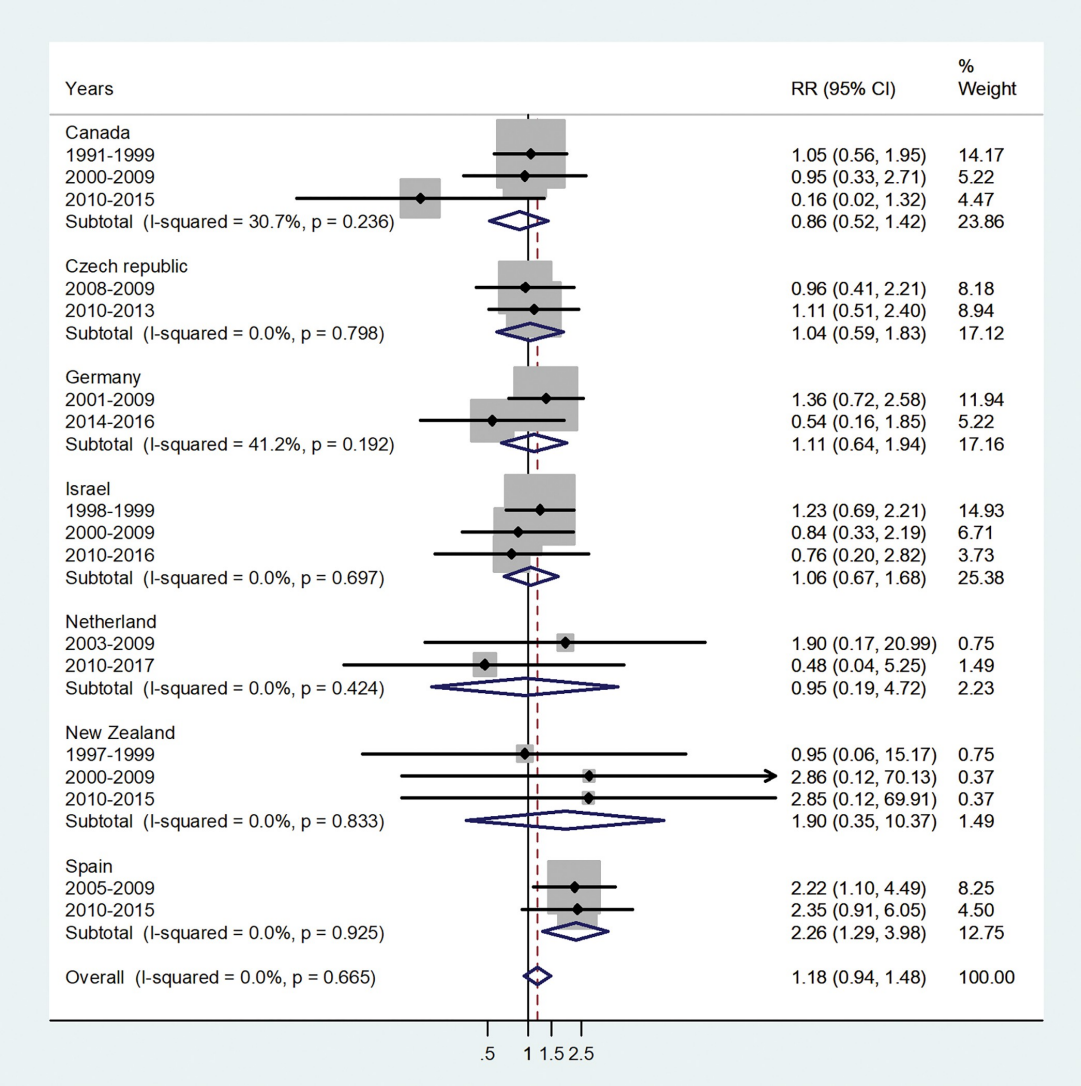
Forest plot of the male to female hepatitis A incidence rate ratios (IRR) in infancy for different years in Canada, Czech Republic, Germany, Israel, Netherland, New Zealand, and Spain.

**Fig 2:**
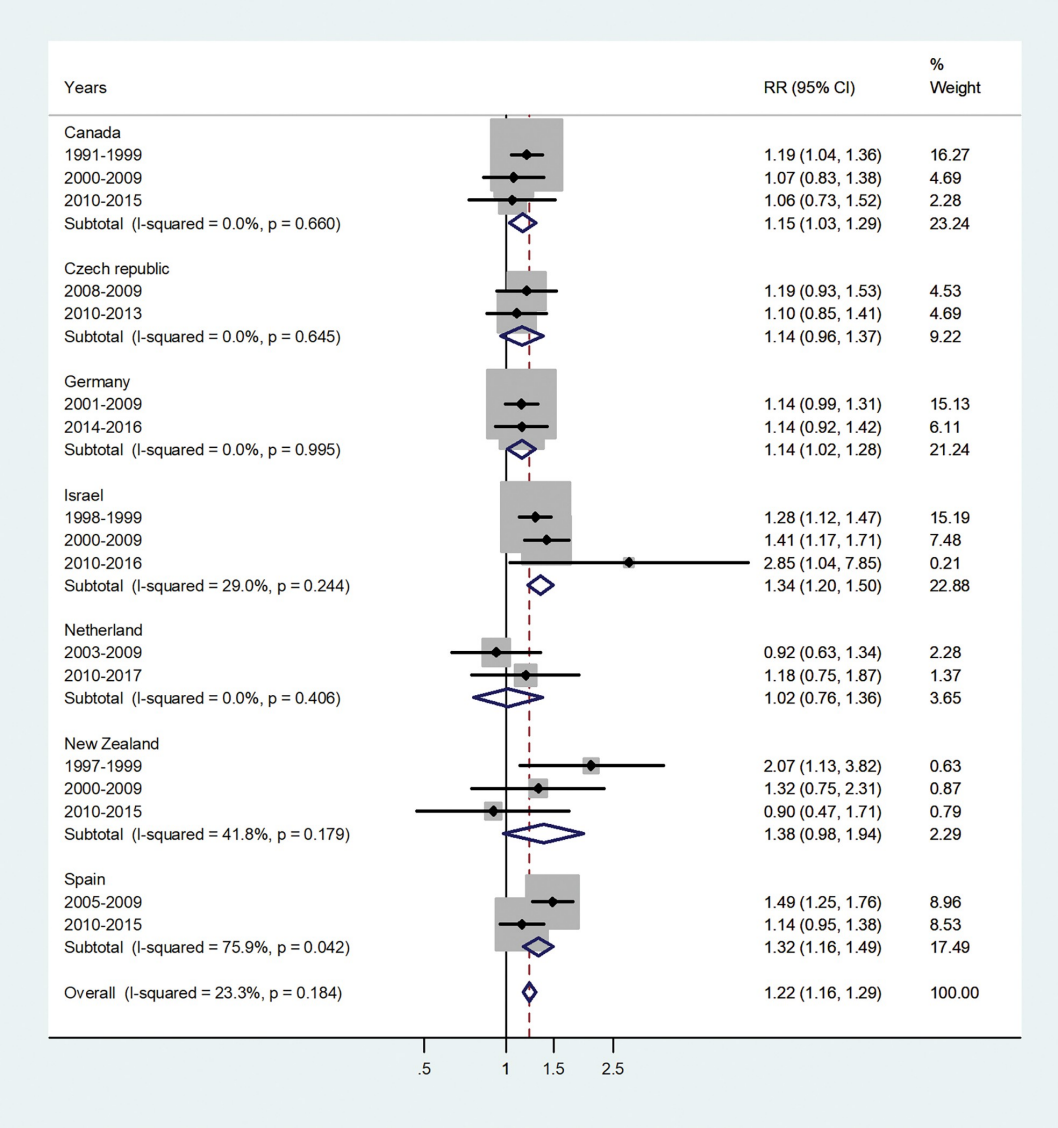
Forest plot of the male to female hepatitis A incidence rate ratios (IRR) at age 1–4, for different years in Canada, Czech Republic, Germany, Israel, Netherland, New Zealand, and Spain.

**Fig 3:**
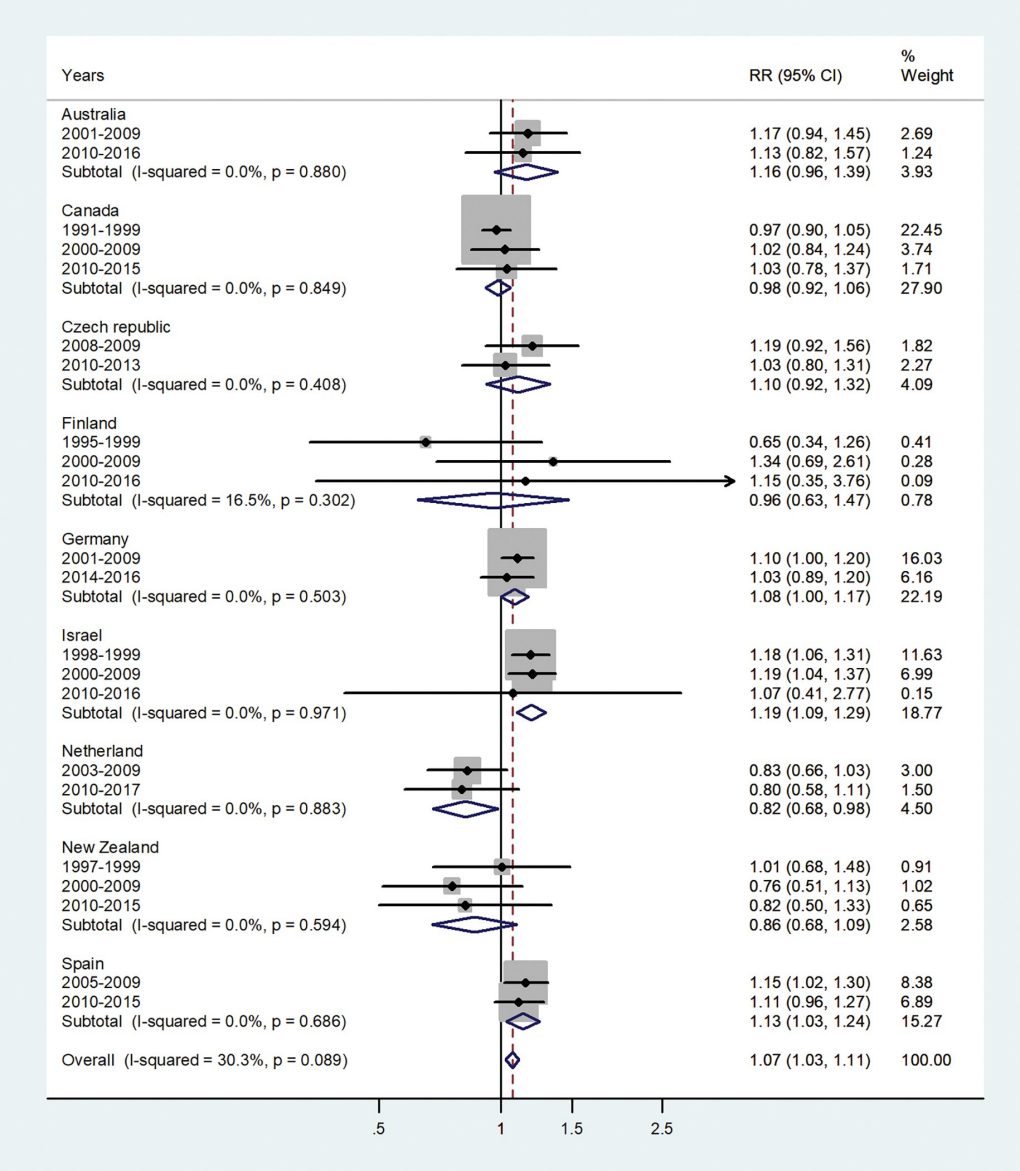
Forest plot of the male to female hepatitis A incidence rate ratios (IRR) at age 5–9, for different years in Australia, Canada, Czech Republic, Finland, Germany, Israel, Netherland, New Zealand, and Spain.

**Fig 4:**
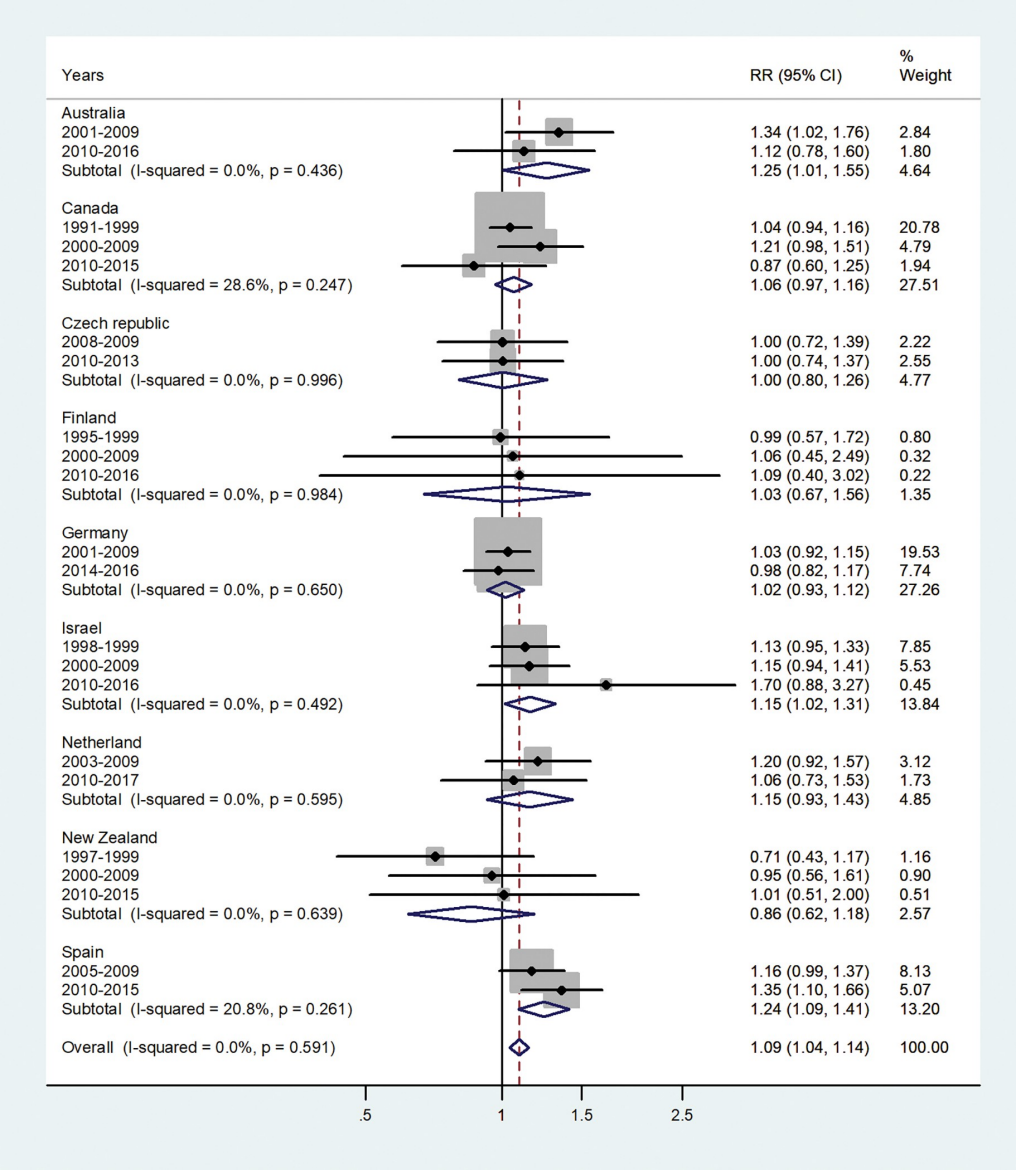
Forest plot of the male to female hepatitis A incidence rate ratios (IRR) at age 10–14, for different years in Australia, Canada, Czech Republic, Finland, Germany, Israel, Netherland, New Zealand, and Spain.

**Fig 5:**
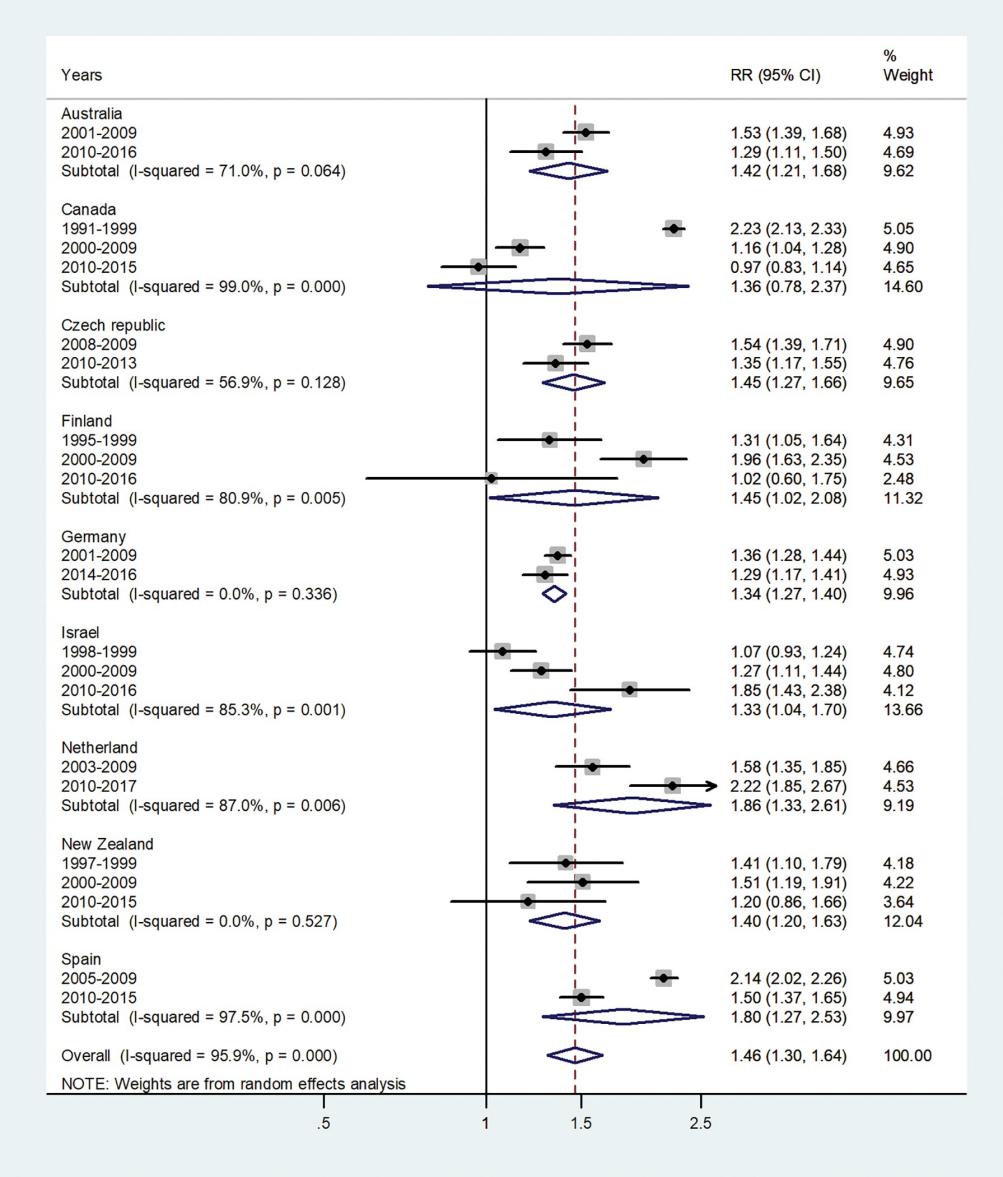
Forest plot of the male to female hepatitis A incidence rate ratios (IRR) at age 15–44 (15–39), for different years in Australia, Canada, Czech Republic, Finland, Germany, Israel, Netherland, New Zealand, and Spain.

**Fig 6:**
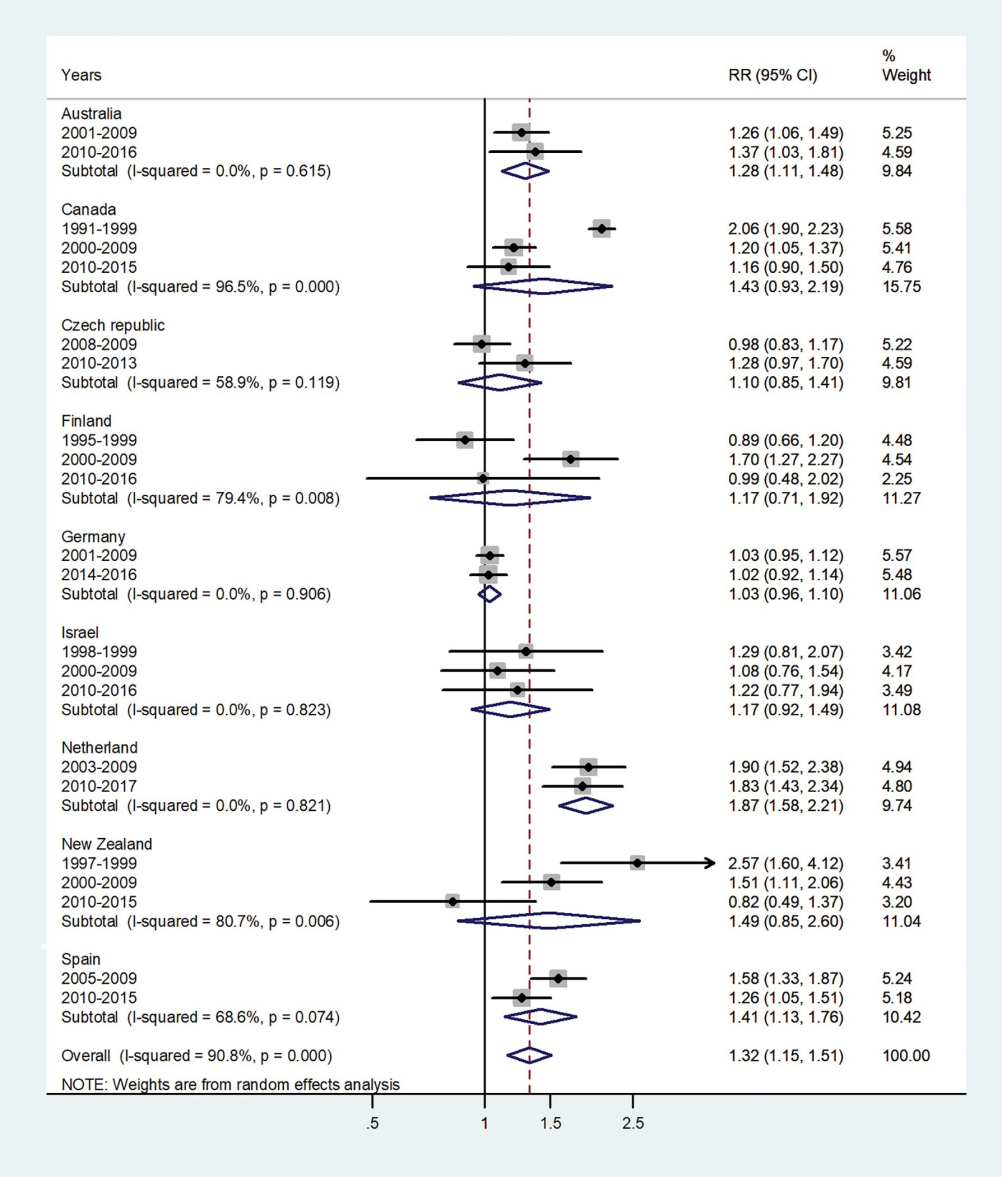
Forest plot of the male to female hepatitis A incidence rate ratios (IRR) at age 45-64(40–59), for different years in Australia, Canada, Czech Republic, Finland, Germany, Israel, Netherland, New Zealand, and Spain.

**Fig 7:**
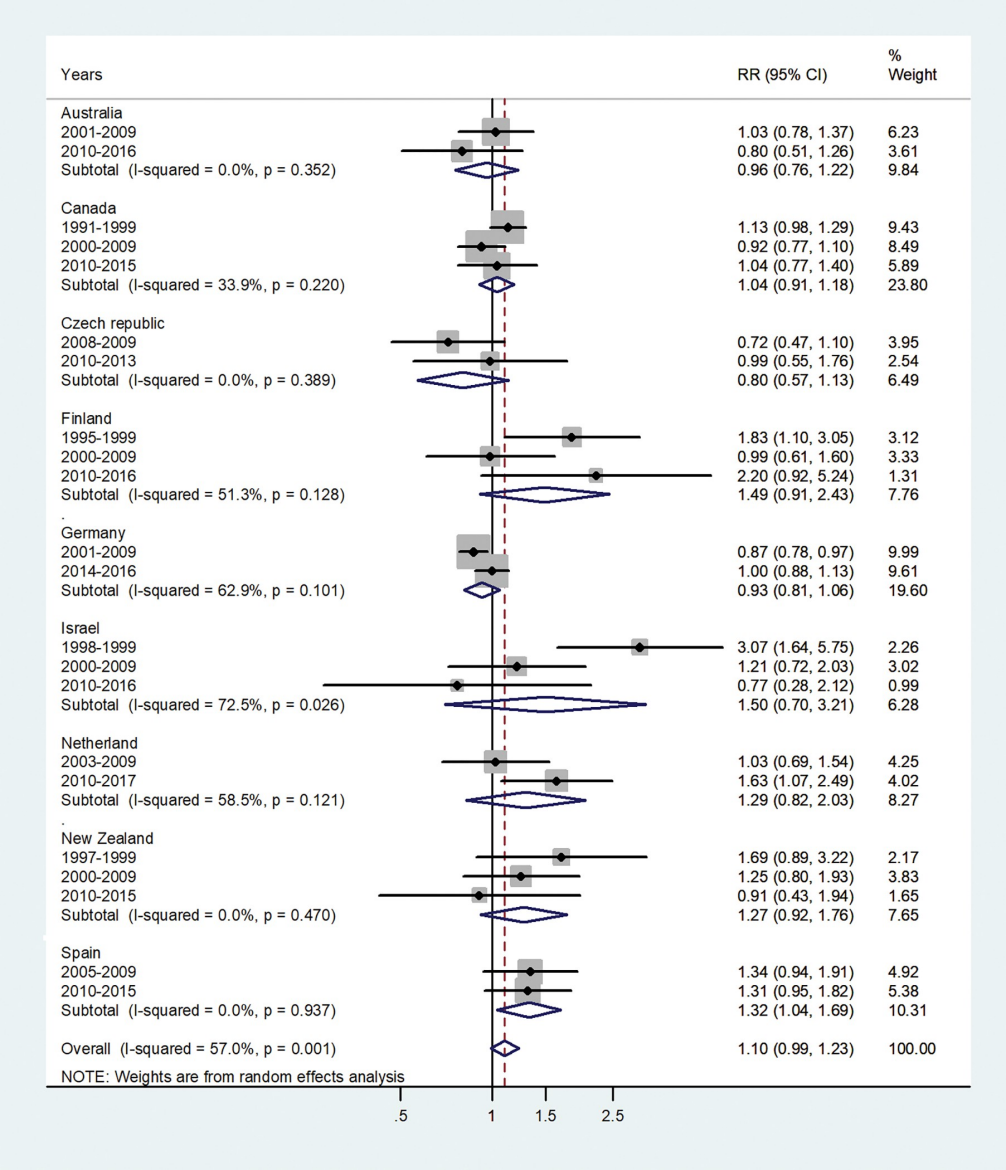
Forest plot of the male to female hepatitis A incidence rate ratios (IRR) at age 65+ (60+), for different years in Australia, Canada, Czech Republic, Finland, Germany, Israel, Netherland, New Zealand, and Spain.

The forest plots for the IRRs by age group, are shown in Figs 1–7.

The forest plot for infants is shown in Fig 1. The pooled male to female IRR was 1.18 (95% CI 0.94–1.48) with I^2^ = 0.0% and varied between 0.86 in Canada and 2.26 in Spain.

The forest plot for the age 1–4 is shown in Fig 2. The pooled IRR was 1.22 (95% CI 1.16–1.29) with I^2^ = 23.3% and varied from 1.02 in Netherland 1.38 in New Zealand.

The forest plot for age 5–9 is given in Fig 3. The pooled IRR was 1.07 (95% CI 1.03–1.11) with I^2^ = 30.3% and varied from 0.82 in the Netherlands to 1.19 in Israel.

The forest plot for age 10–14 is given in Fig 4. The pooled IRR was 1.09 (95% CI 1.04–1.14) with I^2^ = 0.0% and varied between 0.86 in New Zealand to 1.25 in Australia.

The forest plot for age 15–44 is given in Fig 5. The pooled IRR was 1.46 (95% CI 1.30–1.64), I^2^ = 95.9% and varied between 1.33 in Israel to 1.86 in the Netherlands.

The forest plot for age 45–64 is shown in Fig 6. The pooled IRR = 1.32 (95% CI 1.15–1.51), I^2^ = 90.8%, and varied from 1.03 in Germany to 1.87 in the Netherlands.

The forest plot for age 65+ is given in Fig 7. The pooled IRR was 1.10 (95% CI 0.99–1.23) I^2^ = 57.0% and varied from 0.80 in Czech Republic to 1.50 in Israel.

#### Other analyses

Meta-regression analysis showed that almost all the variance in the incidence RRs was contributed by the age groups, with small differences between countries and time periods. To evaluate the effect of individual countries on the male to female incidence ratios, we performed leave-one-out sensitivity analysis and recomputed the pooled IRRs (presented in Tables 2 and 3).

**Table 2:** Sensitivity analysis, by age group and country.

Sensitivity by country							
Countries Removed	Infants	Early childhood	Late childhood	Puberty	Young adulthood	Middle adulthood	Senior adulthood
**Australia**	-	-	1.04 (0.96–1.13)	1.08 (1.03–1.14)	1.58 (1.37–1.82)	1.35 (1.11–1.64)	1.13 (0.98–1.3)
**Canada**	1.29 (0.99–1.66)	1.24 (1.17–1.32)	1.06 (0.98–1.15)	1.1 (1.04–1.16)	1.52 (1.33–1.74)	1.3 (1.11–1.51)	1.13 (0.96–1.34)
**Czech Republic**	1.21 (0.95–1.56)	1.23 (1.16–1.3)	1.04 (0.96–1.13)	1.09 (1.04–1.15)	1.58 (1.37–1.81)	1.39 (1.15–1.67)	1.14 (1–1.29)
**Finland**	-	-	1.05 (0.97–1.14)	1.09 (1.04–1.14)	1.56 (1.36–1.79)	1.36 (1.12–1.64)	1.08 (0.95–1.23)
**Germany**	1.2 (0.93–1.54)	1.24 (1.17–1.32)	1.04 (0.95–1.14)	1.12 (1.06–1.18)	1.6 (1.41–1.81)	1.4 (1.2–1.62)	1.15 (1–1.32)
**Israel**	1.23 (0.94–1.59)	1.19 (1.12–1.26)	1.03 (0.95–1.11)	1.08 (1.02–1.14)	1.61 (1.42–1.83)	1.36 (1.13–1.65)	1.07 (0.95–1.2)
**Netherland**	1.19 (0.94–1.5)	1.23 (1.17–1.3)	1.08 (1.01–1.15)	1.09 (1.03–1.14)	1.54 (1.34–1.76)	1.29 (1.07–1.55)	1.09 (0.95–1.24)
**New Zealand**	1.17 (0.93–1.47)	1.22 (1.16–1.29)	1.08 (1.01–1.15)	1.1 (1.04–1.15)	1.59 (1.38–1.81)	1.32 (1.09–1.6)	1.09 (0.96–1.25)
**Spain**	1.02 (0.8–1.32)	1.2 (1.13–1.28)	1.08 (1.01–1.15)	1.07 (1.01–1.12)	1.52 (1.33–1.74)	1.33 (1.09–1.63)	1.08 (0.95–1.22)

IRR = Incidence rate ratio; CI = confidence interval

**Table 3:** Sensitivity analysis, by age group and years.

Sensitivity by years							
Years Removed	Infants	Early childhood	Late childhood	Puberty	Young adulthood	Middle adulthood	Senior adulthood
**1991–1999**	1.2 (0.92–1.58)	1.21 (1.13–1.29)	1.08 (1.03–1.13)	1.11 (1.05–1.17)	1.49 (1.3–1.72)	1.19 (1.14–1.24)	0.97 (0.87–1.09)
**2000–2009**	1.07 (0.79–1.45)	1.21 (1.12–1.29)	1.04 (0.99–1.09)	1.07 (1–1.14)	1.68 (1.16–2.43)	1.51 (0.92–2.48)	1.13 (0.94–1.35)
**2010–2017**	1.26 (0.96–1.64)	1.25 (1.18–1.33)	1.07 (1.03–1.12)	1.09 (1.03–1.15)	1.8 (1.43–2.27)	1.52 (0.94–2.46)	1.06 (0.79–1.42)

IRR = Incidence rate ratio; CI = confidence interval

After omitting each country (one country at a time, Table 2) or a group of years at a time (Table 3), the pooled IRR’s remained very similar.

Thus, no single country or group of years substantially affected the pooled IRRs. This confirms that the results of this pooled analysis are stable and robust.

## Discussion

In this study, we found that the incidence rates of clinically manifested HAV, pooled over a number of years, for various high-income countries, are consistently higher in males in all age groups. In the youngest and oldest age groups, where the numbers were small, the confidence intervals included unity. Based on the pooled analysis of national data from nine countries, over a period of 6–25 years, we found that the incidence rates of clinical hepatitis A were higher in males by 22%, 7%, 9%, 46%, 32%, and 10% in the age groups 1–4, 5–9, 10–14, 15–44, 45–64 and 65+ respectively.

While sex differences in the incidence of HAV have been examined in a number of studies, they have usually been conducted in individual countries or selected groups of patients. For example, in a national study in Israel in 1992, there was a male predominance of HAV incidence rates [30]. This sex differential was especially pronounced among infants. In a 15-year nationwide epidemiological study in Taiwan, there were higher hospitalization rates in males while male sex and age over 40 years were significant factors associated with mortality [31]. In the study of HAV patients in Saudi Arabia, no sex differences were among hospitalized patients [12]. In a hepatitis A outbreak in Chiba, Japan, in 2011, 40.7% of the 27 patients were male [32], and in another, 65% of the 60 patients were male [33]. However, these figures may simply represent gender differences in exposure to the virus. In addition, the impact of vaccines on sex differences in HAV incidence rates is not clear. There is evidence that females may respond with up to 2–3 times higher anti-HAV antibody levels than males after the priming and after the booster dose and has been observed at different ages [34–37].

The incidence of both viral and bacterial diseases have frequently been reported to be higher in males [1–3]. In addition, there are reported sex differences in the severity of different infections, suggesting that males are more prone to suffer from clinical manifestations of infections than females [38, 39]. While in excess morbidity in males is most common for infectious diseases [1–3], pertussis is a prominent exception, where there is a female excess in morbidity [40]. It is of interest that in the COVID-19 pandemic, there has been no clear evidence of sex differences in incidence rates, although case-fatality rates have consistently been reported to be higher in males [41, 42], even after controlling for other variables.

It has been shown that the male to female IRRs differential will be most evident where there is a low proportion of clinical disease [30]. Since children more commonly suffer from asymptomatic HAV infection [43, 44] and the clinical to subclinical ratio for HAV increases with age, one might expect that the male excess in disease would be less evident at older ages. However, the higher male to female IRRs in the older age groups is most likely due to larger differences in exposure in high risk groups such as in the men who have sex with men (MSM) or people who are HIV positive [7, 8, 45–49]. Thus, behavioral factors can partially explain sex differences in HAV incidence rates in the older age groups. For the youngets age group, there may be protection from maternal HAV antibodies on short-term immunity [50]. However, we have not found evidence that it impacts male and female infants differently.

The exact mechanisms underlying the excess HAV incidence rates in males found in this study are not clear and probably multi-factorial. This study was not designed to address the mechanisms. In addition to behavioral differences, genetic and hormonal factors could be important. In infants and early childhood, and based on the seroprevalence studies, it is unlikely that the sex differences in incidence rates are due to differences in exposure [51]. A study of kindergarten children showed that females had higher anti-HAV antibodies than males [52]. In adults, the results are varied. In a study of blood donors in the US in 2015, [53] no sex differences were observed in the prevalence of anti-HAV IgG antibodies (61% and 60% for males and females, respectively). In a study of ambulatory patients in Portugal between 2002 and 2012, no significant differences between sexes were observed [54]. In a study of refugees and asylum seekers in Germany, HAV seroprevalence rates were higher in adult males than females [55].

Although liver injury in hepatitis A is known to be caused by immune-mediated events, the exact biological mechanisms are not clarified. It is plausible that immune-related mechanisms of liver injury are common to the pathogenesis of all types of hepatitis [56]. Virus-specific CD8+ T cells from hepatitis A patients are considered as a major cause of liver damage. Natural killer cells are also involved and contribute to liver damage [57, 58]. In hepatitis A patients, serum levels of cytokines and chemokines, including interleukin (IL)-6, IL-8, IL-18, IL-22, CXC-chemokine ligand (CXCL)9, and CXCL10 are increased [59] and contribute to liver injury. Many studies have shown that the overall inflammatory response, innate and adaptive immune systems are stronger in females than males, with greater CD4+ T-cell counts a higher CD4+ /CD8+ ratio in females but higher CD8+ T and NK frequencies in males [60].

Sex differences in the clinical expression of hepatitis A may be related to the imbalance in the expression of genes encoded on the X and Y-chromosomes of a host. X chromosome‐associated biological processes and X‐linked genes are responsible for the immunological advantage of females due to the X‐linked microRNAs related processes. The phenomenon of X chromosome inheritance and expression is a cause of immune disadvantage of males and the enhanced survival of females following immunological challenges [61].

The increase in sex hormone levels in infancy that mimics sex steroid levels during puberty (‘minipuberty’) could affect immune cells differently in boys and girls. Testosterone levels predominate in boys at 1–3 months of age and decline at 6–9 months of age, whereas in girls, estradiol levels remain elevated longer [62]. This phenomenon of ’’mini-puberty’’ with sex differences in gonadal hormone levels could influence the maturation of the immune system [63]. This transient rise in sex steroid levels may also influence immune cells differently between boys and girls at later ages [64]. Before any physical signs of puberty, girls had higher levels of estrogens than boys at age 5–9. These higher estradiol levels or lower testosterone levels in young girls may play a part in protection against clinical disease and should be investigated further.

### Strengths and limitations

This current study has several strengths and limitations. The inclusion of nine countries, each evaluated over a number of years, allowed us to evaluate the consistency of the findings over different populations and many years. The analyses are based on national data where both the numbers of cases and denominators are large. Selection bias has been minimized by using national data, which should be representative of each country. However, the countries evaluated in this study are classified as high-income, so the results may not be directly generalizable to low- and middle-income countries. Differential underreporting between countries is likely and may contribute to the variability in the incidence of reported cases of HAV. However, there does not appear to be any reason to believe that the reporting differs between males and females. In the countries examined, there is no evidence that male infants and children are more likely to receive health care. Thus any information bias in the underreporting of incidence rates will most likely be non-differential by sex and the IRRs should not be materially affected. In adults, there could be gender differences in the utilization of medical care, although reports suggest that females in some countries tend to make greater use of health services [65], which would operate in the opposite direction of our observations.

## Conclusions

This study provides stable estimates of the excess male incidence rates in hepatitis A incidence rates in most age groups. While much of the excess in older males may be attributed to differential exposure, the excess in young males, while not large, is remarkably consistent over a number of high-income countries and for extended periods of time. The mechanism is largely unknown. A better understanding of the gender differences can help to elucidate genetic and hormonal determinants of HAV infection and contribute to the role of sex as a biological variable.

## References

[1] Green MS, Schwartz N, Peer V. Sex differences in campylobacteriosis incidence rates at different ages—a seven country, multi-year, meta-analysis. A potential mechanism for the infection. BMC Infect Dis. 2020;20:625. doi: 10.1186/s12879-020-05351-6 32842973 PMC7445732

[2] Peer V, Schwartz N, Green MS. Consistent, excess viral meningitis incidence rates in young males: A multi-country, multi-year, meta-analysis of national data. The importance of sex as a biological variable. EClinicalMedicine. 2019;15:62–71. doi: 10.1016/j.eclinm.2019.08.006 31709415 PMC6833362

[3] Peer V, Schwartz N, Green MS. A pooled analysis of sex differences in rotaviral enteritis incidence rates in three countries over different time periods. Womens Health Rep (New Rochelle). 2022; 3:228–237 doi: 10.1089/whr.2021.0096 35262061 PMC8896211

[4] Jacobsen KH. Globalization and the changing epidemiology of hepatitis A virus. Cold Spring Harb Perspect Med. 2018;8:a031716. doi: 10.1101/cshperspect.a031716 29500305 PMC6169986

[5] Zeng Dan-Yi, Li Jing-Mao, Lin Su, et al. Global burden of acute viral hepatitis and its association with socioeconomic development status, 1990–2019. J Hepatol. 2021. 75:547–556. doi: 10.1016/j.jhep.2021.04.035 33961940

[6] Green MS, Block C, Slater PE. Rise in the incidence of viral hepatitis in Israel despite improved socioeconomic conditions. Rev Infect Dis. 1989; 11:464–469. doi: 10.1093/clinids/11.3.464 2749104

[7] Alberts CJ, Boyd A, Bruisten SM, et al. Hepatitis A incidence, seroprevalence, and vaccination decision among MSM in Amsterdam, the Netherlands. Vaccine. 2019;37:2849–2856 doi: 10.1016/j.vaccine.2019.03.048 30992222

[8] Honda M, Asakura H, Kanda T, et. al. Male-dominant hepatitis A outbreak observed among non-HIV-infected persons in the northern part of Tokyo, Japan.Viruses. 2021;13:207. 33573054 10.3390/v13020207 PMC7910831

[9] Andani A, Bunge E, Kassianos G, et.al. Hepatitis A occurrence and outbreaks in Europe over the past two decades: a systematic review. J Viral Hepat. 2023 10.1111/jvh.13821 36825922

[10] Bauer D, Farthofer A, Chromy D, et al. Recent outbreaks of severe hepatitis A virus infections in Vienna. Eur J Clin Microbiol Infect Dis. 2021;40:335–344. doi: 10.1007/s10096-020-04028-x 32940811 PMC7817601

[11] Dudareva S, Faber M, Zimmermann R, et.al. Epidemiology of viral hepatitis A to E in Germany. Bundesgesundheitsblatt Gesundheitsforschung Gesundheitsschutz. 2022;65:149–158. 35029725 10.1007/s00103-021-03478-8 PMC8758919

[12] Al-Tawfiq JA, Anani A. Profile of viral hepatitis A, B, and C in a Saudi Arabian hospital. Med Sci Monit. 2008;14:CR52–56. 18160946

[13] Choe YJ, Son H. The changing gender differences in hepatitis A incidence in South Korea. Vaccine. 2020;38: 712–714. doi: 10.1016/j.vaccine.2019.11.048 31787416

[14] National Notifiable Diseases Surveillance System (NNDSS), Department of Health. Available at: http://www9.health.gov.au/cda/source/rpt_5_sel.cfm. Accessed 1 April 2018.

[15] Public Health Agency of Canada. Available at: https://www.canada.ca/en/public-health.html. Accessed on 1 June 2018.

[16] Institute of Health Information and Statistics. Available at: https://www.uzis.cz/en/catalogue/infectious-diseases. Accessed 1 March 2018.

[17] National institute for health and welfare (THL): https://www.thl.fi/ttr/gen/rpt/tilastot.html. Accessed on May 1, 2018.

[18] German Federal Health Monitoring System. Available at: http://www.gbe-bund.de/ gbe10/pkg_isgbe5.prc_isgbe? p_uid¼gast&p_aid¼0&p_sprache¼D (1 February 2018, date last accessed).

[19] Environmental Science and Research (ESR) for the Ministry of Health. Available at: https://surv.esr.cri.nz/surveillance/annual_surveillance.php. Accessed 30 March 2018.

[20] Instituto de Salud Carlos III. Available: http://www.eng.isciii.es/ISCIII/es/contenidos/fd-servicios-cientifico-tecnicos/fd-vigilancias-alertas/fd-enfermedades/enfermedades-declaracion-obligatoria-informes-anuales.shtml. Accessed 1 March 2018.

[21] ABS.Stat (Australian Bureau of Statistics). Available at: http://stat.data.abs.gov.au/Index.aspx?DatasetCode=ABS_ERP_ASGS2016.Accessed 15 May 2018.

[22] Statistics, Canada, CANSIM database: Available at: https://www150.statcan.gc.ca/t1/tbl1/en/cv.action?pid=1710010201. Accessed 1 June 2018.

[23] Czech Statistical Office. Available at: https://www.czso.cz/csu/czso/population. Accessed 1 March 2018.

[24] Statistics Finland’s PX-Web databases. Available at: http://pxnet2.stat.fi/PXWeb/pxweb/en/StatFin/StatFin__vrm__vaerak/statfin_vaerak_pxt_021.px/?rxid=2f968705-bdaa-48b1-9d5a-d4985ead7d40. Accessed 15 April 2018.

[25] German Federal Health Monitoring System: http://www.gbe-bund.de/gbe10/abrechnung.prc_abr_test_logon?p_uid=gast&p_aid=46300054&p_knoten=VR&p_sprache=E&p_suchstring=population. Accessed on February 1, 2018

[26] Central Bureau of Statistics: http://www.cbs.gov.il/reader/shnatonhnew_site.htm?sss=%E4%EE%F9%EA&shnaton_scan=45. Accessed on March 1, 2018.

[27] Statistics Netherlands’ database (StatLine). Available at: https://opendata.cbs.nl/statline/#/CBS/en/dataset/37325eng/table?ts=1528798782913. Accessed 15 May 2018.

[28] Stats NZ, Infoshare. Available at: http://archive.stats.govt.nz/infoshare/SelectVariables.aspx?pxID=b854d8a2-3fdf-402c-af69-604112e80baa. Accessed 15 May 2018.

[29] Demographic Statistics Database (United Nations Statistics: Division). Available at: http://data.un.org/Data.aspx?d=POP&f=tableCode%3A22. Accessed 1 April 2018.

[30] Green MS. The male predominance in the incidence of infectious diseases in children: a postulated explanation for disparities in the literature. Int J Epidemiol. 1992;21:381–386 doi: 10.1093/ije/21.2.381 1428496

[31] Chen CM, Chen SC, Yang HY, Yang ST, Wang CM. Hospitalization and mortality due to hepatitis A in Taiwan: a 15-year nationwide cohort study. J Viral Hepat. 2016;23:940–945. doi: 10.1111/jvh.12564 27386835

[32] Tominaga A, Kanda T, Akiike T, et.al. Hepatitis A outbreak associated with a revolving sushi bar in Chiba, Japan: Application of molecular epidemiology. Hepatol Res. 2012;42: 828–834. doi: 10.1111/j.1872-034X.2012.00988.x 22776552

[33] Takahashi H, Yotsuyanagi H, Yasuda K, et.al. Molecular epidemiology of hepatitis A virus in metropolitan areas in Japan. J Gastroenterol. 2006;41:981–986. doi: 10.1007/s00535-006-1888-9 17096067

[34] Bovier PA, Bock J, Loutan L, Farinelli T, Glueck R, Herzog C. Long-term immunogenicity of an inactivated virosome hepatitis A vaccine. J Med Virol. 2002; 68:489–493. doi: 10.1002/jmv.10244 12376955

[35] Höhler T, Groeger-Bicanic G, Hoet B, Stoffel M. Antibody persistence and immune memory elicited by combined hepatitis A and B vaccination in older adults. Vaccine. 2007; 25:1503–1508. doi: 10.1016/j.vaccine.2006.10.024 17097774

[36] Spradling PR, Bulkow LR, Negus SE, Homan C, Bruce MG, McMahon BJ. Persistence of seropositivity among persons vaccinated for hepatitis A during infancy by maternal antibody status: 15-year follow-up. Hepatology. 2016; 63:703–711. doi: 10.1002/hep.28375 26637987 PMC6459008

[37] Van Herck K, Hens A, De Coster I, et.al. Long-term antibody persistence in children after vaccination with the pediatric formulation of an aluminum-free virosomal hepatitis A vaccine. Pediatr Infect Dis J. 2015; 34:e85–91 doi: 10.1097/INF.0000000000000616 25389920

[38] Klein SL, Flanagan KL. Sex differences in immune responses. Nat Rev Immunol. 2016;16:626–638 doi: 10.1038/nri.2016.90 27546235

[39] Klein SL, Jedlicka A, Pekosz A. The Xs and Y of immune responses to viral vaccines. Lancet Infect Dis. 2010;10:338–349. doi: 10.1016/S1473-3099(10)70049-9 20417416 PMC6467501

[40] Peer V, Schwartz N, Green MS. A multi-country, multi-year, meta-analytic evaluation of the sex differences in age-specific pertussis incidence rates. PLoS One. 2020;15:e0231570 doi: 10.1371/journal.pone.0231570 32324790 PMC7179848

[41] Jacobsen H, Klein SL. Sex differences in immunity to viral Infections. Front Immunol. 2021;12:720952 doi: 10.3389/fimmu.2021.720952 34531867 PMC8438138

[42] Green MS, Nitzan D, Schwartz N, Niv Y, Peer V. Sex differences in the case-fatality rates for COVID-19-A comparison of the age-related differences and consistency over seven countries. PLoS One. 2021;16(4):e0250523 doi: 10.1371/journal.pone.0250523 33914806 PMC8084161

[43] Wensley A, Smout E, Ngui SL, et.al. An outbreak of hepatitis A virus infection in a secondary school in England with no undetected asymptomatic transmission among students. Epidemiol Infect. 2022; 151:e6 doi: 10.1017/S095026882200190X 36502811 PMC9990387

[44] Abutaleb A, Kottilil S. Hepatitis A: epidemiology, natural history, unusual clinical manifestations, and prevention. Gastroenterol Clin North Am. 2020;49:191–199. doi: 10.1016/j.gtc.2020.01.002 32389358 PMC7883407

[45] Lin KY, Chen GJ, Lee YL, et.al. Hepatitis A virus infection and hepatitis A vaccination in human immunodeficiency virus-positive patients: A review. World J Gastroenterol. 2017; 23: 3589–3606. doi: 10.3748/wjg.v23.i20.3589 28611512 PMC5449416

[46] Franco E, Giambi C, Ialacci R, Coppola RC, Zanetti AR. Risk groups for hepatitis A virus infection. Vaccine. 2003;21:2224–2233 doi: 10.1016/s0264-410x(03)00137-3 12744847

[47] Migueres M, Lhomme S, Izopet J. Hepatitis A: epidemiology, high-risk groups, prevention and research on antiviral treatment. Viruses. 2021;13:1900 doi: 10.3390/v13101900 34696330 PMC8540458

[48] Yoshimura Y, Horiuchi H, Sawaki K, et.al. Hepatitis A Outbreak Among Men Who Have Sex With Men, Yokohama, Japan, January to May 2018. Sex Transm Dis. 2019;46: e26–e27. doi: 10.1097/OLQ.0000000000000937 30395105

[49] Bazzardi R, Dore E, Ciccozzi M, et.al. Outbreak of acute hepatitis A associated with men who have sex with men (MSM) in North Sardinia 2017–2018. J Infect Dev Ctries. 2020;14:1065–1070. doi: 10.3855/jidc.12184 33031097

[50] Bell BP, Negus S, Fiore AE, et.al. Immunogenicity of an inactivated hepatitis A vaccine in infants and young children. J.Pediatr Infect Dis J. 2007;26: 116–122. doi: 10.1097/01.inf.0000253253.85640.cc 17259872

[51] Walter F, Ott JJ, Claus H, Krause G. Sex- and age patterns in incidence of infectious diseases in Germany: analyses of surveillance records over a 13-year period (2001–2013). Epidemiol Infect. 2018;146: 372–378. doi: 10.1017/S0950268817002771 29357958 PMC9134510

[52] Lin DB, Tsai TP, Yang CC, et.al. Association between seropositivity of antibodies against hepatitis a virus and Helicobacter pylori. Am J Trop Med Hyg. 2000;63:189–191. doi: 10.4269/ajtmh.2000.63.189 11388513

[53] Tejada-Strop A, Zafrullah M, Kamili S, Stramer SL, Purdy MA. Distribution of hepatitis A antibodies in US blood donors. Transfusion. 2018;58: 2761–2765. doi: 10.1111/trf.14916 30284286 PMC6283683

[54] Pereira S, Linhares I, Neves AF, Almeida A. Hepatitis A immunity in the District of Aveiro (Portugal): an eleven-year surveillance study (2002–2012). Viruses. 2014;6: 133613–45. doi: 10.3390/v6031336 24638206 PMC3970153

[55] Jablonka A, Solbach P, Wöbse M, et.al. Seroprevalence of antibodies and antigens against hepatitis A-E viruses in refugees and asylum seekers in Germany in 2015. Eur J Gastroenterol Hepatol. 2017;29: 939–945. doi: 10.1097/MEG.0000000000000889 28492419

[56] Shin EC, Sung PS, Park SH. 2016a. Immune responses and immunopathology in acute and chronic viral hepatitis. Nat Rev Immunol. 2016: 509–523. doi: 10.1038/nri.2016.69 27374637

[57] Wang M, Feng Z. Mechanisms of Hepatocellular Injury in Hepatitis A. Viruses. 2021;13:861. doi: 10.3390/v13050861 34066709 PMC8151331

[58] Vallbracht A, Maier K, Stierhof YD, Wiedmann KH, Flehmig B, Fleischer B. Liver-derived cytotoxic T cells in hepatitis A virus infection. J Infect Dis. 1989;160:209–217. doi: 10.1093/infdis/160.2.209 2503564

[59] Shin SY, Jeong SH, Sung PS, et.al. Comparative analysis of liver injury-associated cytokines in acute hepatitis A and B. Yonsei Med J. 2016;57:652–657. doi: 10.3349/ymj.2016.57.3.652 26996565 PMC4800355

[60] Abdullah M, Chai P-S, Chong M-Y, et al. Gender effect on in vitro lymphocyte subset levels of healthy individuals. Cell Immunol. 2012; 272:214–219. doi: 10.1016/j.cellimm.2011.10.009 22078320

[61] Schurz H, Salie M, Tromp G, Hoal EG, Kinnear CJ, Möller M. The X chromosome and sex-specific effects in infectious disease susceptibility. Hum Genomics. 2019;13:2. doi: 10.1186/s40246-018-0185-z 30621780 PMC6325731

[62] Lanciotti L, Cofini M, Leonardi A, Penta L, Esposito S. Up-To-Date review about minipuberty and overview on hypothalamic-pituitary-gonadal axis activation in fetal and neonatal life. Front Endocrinol (Lausanne). 2018;9:410. doi: 10.3389/fendo.2018.00410 30093882 PMC6070773

[63] Moreira-Filho CA, Bando SY, Bertonha FB, et.al Minipuberty and sexual dimorphism in the infant human thymus. Sci Rep. 2018;8: 13169. doi: 10.1038/s41598-018-31583-3 30177771 PMC6120939

[64] Courant F, Aksglaede L, Antignac JP, et.al. Assessment of circulating sex steroid levels in prepubertal and pubertal boys and girls by a novel ultrasensitive gas chromatography-tandem mass spectrometry method. J Clin Endocrinol Metab. 2010;95:82–92. doi: 10.1210/jc.2009-1140 19933393

[65] Bertakis KD, Azari R, Helms LJ, Callahan EJ, Robbins JA. Gender differences in the utilization of health care services. J Fam Pract. 2000;49:147–152. 10718692

